# Letter to the editor re: the CDC blood lead reference value for children

**DOI:** 10.1186/s12940-019-0472-8

**Published:** 2019-04-11

**Authors:** Perry Gottesfeld, Deborah A. Cory-Slechta

**Affiliations:** 1 Occupational Knowledge International, 4444 Geary Boulevard, Suite 208, San Francisco, CA 94118 USA; 2 Department of Environmental Medicine, Box EHSC, University of Rochester Medical Center, Rochester, NY 14642 USA

**Keywords:** Lead, Blood lead

## To the Editor:

Although we completely agree with Paulson and Brown that primary prevention to eliminate sources of exposure is the best long-term approach to childhood lead poisoning, it is not a strategy that can replace the need to prioritize individuals and communities that are over-exposed. They argue that the CDC should reject the advice of two of its’ scientific advisory committees that called on the agency to lower the blood lead reference value for children in the U.S. because it would put clinicians in an untenable position as there is no prescribed clinical response [1]. However, by not informing parents that their children are being exposed to lead at levels well in excess of the median, we are preventing parents from recognizing the need to take precautions to reduce environmental lead exposures.

The CDC blood lead reference value serves a dual purpose to inform individual cases (i.e. parents) that a child’s exposure exceeds the norm and it serves as a public health benchmark to inform the public when their communities are over-exposed. This was the criteria that alerted both physicians and the general public in Flint, Michigan and East Chicago, Indiana that there was reason to be concerned about exposures to lead in these communities. Reports of children’s BLLs above levels previously considered acceptable, resulted in governmental responses to address lead in drinking water at the former and to vacate public housing located on a former industrial site at the later. Paulson and Brown do not explain how these situations would have come to light if the CDC had not adopted the reference value approach in 2012 to identify individuals and communities with the highest exposures.

The authors’ main concern is how the reference value will complicate the role of Pediatricians as the messenger charged with informing parents that a child has an elevated blood lead level for which there is no effective clinical response. But this is not a new role for physicians who are often tasked with communicating public health information derived from epidemiological studies to advise individual patients to consider life style responses. Pediatricians already discuss a range of environmental risk factors with parents including screen time, nutrition, and car seats for which they can offer no treatment or cure.

Paulson and Brown suggest that the blood lead reference value triggers clinical “interventions” but fails to explain that the only response recommended by the CDC is to call for more frequent blood lead testing of the individual child and a nutritional assessment to include testing for iron deficiency. Additional actions including environmental inspections and public health case management are not the responsibility of physicians, but are carried out by public health authorities in some jurisdictions.

Although Paulson and Brown raise legitimate concerns about the reproducibility of laboratories and clinical testing equipment for blood lead levels at 3.5 μg/dl, these potential errors will have minimal impact on the interpretation of individual or aggregate community results. Moreover, efforts are already underway to enhance reproducibility of blood lead testing. In practice, it will make little difference to a parent if their child is above the 97.5th, 90th or even 60th percentile of the NHANES blood lead distribution. Concerns around the reporting of false positive results, do not change the fact that even with some laboratory error, children with reported levels > 3.5 μg/dl from a confirmed venous puncture are experiencing exposures that are elevated in relation to the U.S. population median (0.86 μg/dl) [2]. Similarly, potential testing errors will not impede communities from acting on aggregate blood lead testing data to investigate and identify possible sources of lead exposure.

Our greatest concern is that the article incorrectly states that “the recommended interventions have not been shown to reduce blood lead levels once they are elevated” when there are multiple studies that demonstrate that lead abatement reduces exposures over time [3–6]. The evidence provided for this statement refers to an outdated set of recommendations that called for “controlling” lead hazards in housing (at levels we now understand were inadequate) to prevent exposures instead of eliminating such hazards with a permanent response referred to as abatement.

We fully agree with Paulson and Brown that ideally children should not be used to identify lead hazards and that we should minimize environmental exposures. However, it is also important to recognize that a national primary prevention strategy cannot be implemented overnight and therefore CDC should follow the advice of its independent expert committees and adopt a new reference value for this interim period before lead hazards are eliminated. Without tracking BLLs as “elevated” (or not) in relationship to the population norm, communities would never be able to identify the next Flint, Michigan and parents would miss an opportunity to recognize and respond to lead hazards in their homes.

Sincerely.

Perry Gottesfeld.

Deborah A. Cory-Slechta.

Note: The authors co-chaired the subcommittee to advise the Centers for Disease Control and Prevention (CDC) Advisory Committee on Childhood Lead Poisoning Prevention (ACCLPP) on revising the childhood lead poisoning prevention guidelines from 2010 to 2012.
